# Development of Systems and Futures Thinking Skills by Primary Pre-service Teachers for Addressing Epidemics

**DOI:** 10.1007/s11165-023-10097-7

**Published:** 2023-01-16

**Authors:** Araitz Uskola, Blanca Puig

**Affiliations:** 1 Faculty of Education of Bilbao, University of the Basque Country (UPV/EHU), B. Sarriena S/N, 48940 Leioa, Spain; 2 Faculty of Education Sciences, University of Santiago de Compostela (USC), Santiago de Compostela, Spain

**Keywords:** Agency, One health, Futures thinking, Systems thinking, Primary pre-service teachers’ training

## Abstract

Science educators highlight the importance of developing systems thinking (ST) and futures thinking (FT) for students to make decisions and to be active citizens that address socioscientific problems. The dimensions related to FT take in this study were three implied in ST and two in the appropriation of the future. The aim of this study is to analyse the level of FT-related dimensions developed by a group of pre-service elementary teachers and how far different activities designed to foster them were effective. Written explanations presented by the participants about the origin of pandemics and possible ways to prevent them, as well as videos developed by small groups with the goal to present a campaign for avoiding future pandemics, were analysed. Based on the literature, five dimensions and up to four levels of performances were identified. After completing the activities, participants were able to relate the three spheres of the “One Health” notion to explain the causes of pandemics. Moreover, they established complex cause-effect relationships between the different factors, although they only constructed monocausal relationships when proposing measures. Participants improved their performance in anticipating the future and identifying themselves as agents of change. The elaboration of concept maps facilitated the development of components and behaviour ST dimensions, and the design of the campaign allowed participants to identify themselves as agents of change. The use of these strategies in science education can contribute to the development of a citizenry capable of understanding and acting on systems.

## Introduction

Momentum is growing amongst science educators for employing strategies that help students to develop a more complex way of thinking that connects the causes and processes that explain socioscientific issues such as the COVID-19 pandemic, as well as to take responsibility and anticipate the consequences of our actions. Within the needs of education for 2030, the Organization for Economic Co-operation Development (OECD) recommended that students become agents of change, which requires the development of their systems and futures thinking skills. They explained that “To be prepared for the future, individuals have to learn to think and act in a more integrated way, taking into account the interconnections and inter-relations between contradictory or incompatible ideas, logics and positions, from both short- and long-term perspectives. In other words, they have to learn to be systems thinkers” (OECD, 2018, p. 5).

Systems thinking (ST) allows students to address complex problems in science classrooms in a holistic way (Ben-Zvi-Assaraf & Knippels, 2022). In this study, ST is considered a competence that requires identifying the elements that are part of a system**,** as well as the relationships between them (e.g., Ben-Zvi-Assaraf & Orion, 2005; Hmelo-Silver et al., 2017; Hmelo-Silver & Pfeffer, 2004; Mehren et al., 2018; Snapir et al., 2017), establishing cause-effect networks and identifying non-linear relationships and feedback-loops (Hipkins, 2021; Mehren et al., 2018). Besides, ST demands being aware of phenomena such as self-organization and emergence of the system (Hipkins, 2021), taking an “inside the system” perspective (Hipkins, 2021) and deciding how to act on it (Hipkins, 2021; Mehren et al., 2018; Ossimitz, 2000). Students who are able to apply ST are better equipped to be “change agents,” thereby, able to protect the environment, shaping the future in a positive way (OCDE, 2018). To achieve this goal in science instruction, anticipation and futures thinking (FT) skills are necessary. FT implies discovering, examining, and suggesting future scenarios (Vidergor et al., 2018). In the context of science education, Jones et al., (2012) and Levrini et al., (2021) highlighted the need for integrating FT into science curricula, specifically when dealing with socioscientific issues (SSI). Health problems such as epidemics or pandemics caused by zoonosis such as COVID-19 constitute ideal SSI for the development of ST and FT, since they require an analysis of the origin/causes of the problem from diverse perspectives and the development of responsible actions for anticipating and preventing them from spreading among the population.

Many studies analysed ST about natural systems (Hmelo-Silver et al., 2017; Mambrey et al., 2022); however, few have approached it from a FT perspective, analysing the situation, trends, causes, and possible future scenarios (Jones et al., 2012). In the study of Jones et al., (2012), students engaged in FT in diverse SSI and improved in the identification of trends and drivers. However, a ST perspective was lacking. Thus, these researchers appealed for further investigation on FT in science education that addresses the competences to act in the system to achieve the desired future.

This study approaches the development of FT by pre-service teachers via a systemic perspective, addressing a health problem: the origin of epidemics and actions for avoiding them in the future. We seek to make a contribution to a line of research, FT in science education, that deserves further investigation in order to understand how to help students to deal with emerging problems that involve uncertainty.

### Futures and Systems Thinking for Addressing Health Problems

Futures studies investigate trends to make predictions and create possible, probable, and desirable scenarios from which to guide actions in the present (Levrini et al., 2021). These investigations emphasize the importance of an education that helps learners to think critically and creatively about the future and to develop a sense of being an agent to influence it.

Thinking about the future is part of human cognition, much of human behaviour is future oriented (Atance & Meltzoff, 2005). FT studies have increased in several domains of psychology. However, students’ ability to make predictions about their own future states is underexplored. Research into FT and its importance in science education is still scant, with few available studies on education for sustainable development (ESD) (Johanson, 2021; Rieckmann, 2012). FT is a key competence in ESD that can be developed through students’ participation in scenarios that involve decision-making on socio-environmental issues (Johanson, 2021).

Jones et al., (2012) proposed a conceptual framework for the design and implementation of FT-oriented lessons in science classrooms when dealing with SSI. This framework includes (a) the understanding of the current situation, (b) the identification of trends, (c) drivers, (d) the development of possible scenarios, and (e) the selection of the desirable future. Jones et al., (2012) appealed for the inclusion of action related dimensions in the framework for future works.

According to Levrini et al., (2020, 2021), given the complexity of the problems we face, and young people’s threatening view of the future, students need to develop thinking skills that enable them to imagine diverse futures, to think about their futures and the world’s future as interconnected, and to feel they can deal with the global challenges as responsible and active citizens. Considering oneself as an agent of change and action has been a concern for environmental education for a long time. Environmental educators such as Jensen and Schnack, (1997) set the focus on the development of action competency as a main goal of environmental education in contrast to experiences where the aim is merely to raise awareness of environmental problems or to point out what action should be taken. Being a competency, action competency requires the mobilization of knowledge and skills, but also attitudes and values to meet complex demands (Hodson, 2003) in a particular context. It also involves the ability and desire to be a skilled participant (Jensen & Schnack, 1997). Although pedagogical strategies can be diverse, students would benefit from participating in action-oriented activities that promote the application of knowledge, as they would develop action competency and would position themselves as agents. Oliveira et al., (2015) warn against considering environmental agency as just a rational issue and overlooking that agency, classroom discourse, and activities are socio-culturally mediated. Jensen and Schnack, (1997) identified four dimensions of action competency: (1) knowledge and perception of the environmental problem; (2) commitment to solve the problem; (3) a vision for the future without the problem; (4) experiences of action. Thus, FT and agency are notions that are intimately related, as agency implies the idea of projection and anticipation, and FT impacts on learners’ present actions (Cuzzocrea & Mandich, 2016).

Some studies focus on identifying and characterizing the competencies that are necessary to develop FT. Particularly, a Delphi study with ESD experts from two European and three Latin American countries (Rieckmann, 2012) identified 12 competencies for FT that included ST, anticipatory thinking, and critical thinking. These competencies are also present in the dimensions proposed by Levrini et al., (2021) in the framework of a European project aimed to incorporate FT in science education and to analyse how science education can contribute to its development. Levrini et al., (2021) defined and operationalized several dimensions as follows: (a) structural; (b) dynamic, related to ST; (c) related to broadening the vision of possible future scenarios; and (d) related to the approach to the future, which includes the view of agents of change and the perception of the future as near.

In the aforementioned research about FT-oriented lessons in science classrooms when dealing with SSI (Jones et al., 2012), the authors acknowledged that some of their studies had taken few points of view into account and lacked a systemic view of the situation. Despite the growing interest in ST in science education research, most studies focus on natural systems, such as ecosystems (Hmelo-Silver et al., 2017; Mambrey et al., 2022), the human body (Snapir et al., 2017), or geological systems (Ben-Zvi Assaraf & Orion, 2005), rather than on global issues such as health and environmental issues, that also require a holistic view considering the interrelationships of natural and social systems. Those interrelationships are difficult to consider for students and they need scaffolding through discussion of their findings and concept maps (Ben-Zvi-Assaraf & Knippels, 2022). Hofman-Bergholm, (2018) addressed this issue. She refers to ST as the tool needed to develop the holistic thinking required to achieve a sustainable future. According to this perspective, ST is defined as the ability to analyse complex systems and problems from different spheres (political, social, economic, environmental) and scales (local, global) (Wiek et al., 2011). One example is the “One Health” notion, which considers the human–environment-animal system and specifically the interactions between the parts of that system which make animal, environmental, and human health interconnected and interdependent (Food and Agriculture Organization of the United Nations [FAO] et al., 2019).

The present study is aligned with Levrini et al.’s proposal (2020) of helping students to imagine possible future scenarios to develop agency, based on the idea that FT can stimulate learners to act on and anticipate problems, as projecting an image of the future and imagining a better future requires a link between long-term goals and immediate actions (Rieckmann, 2012). The dimensions of FT considered are those related to ST, anticipation of the future and action taking (Jones et al., 2012; Levrini et al., 2021; Rieckmann, 2012). In the case of ST, a system with several spheres is taken (Hofman-Bergholm, 2018), specifically animal, environmental, and human health (FAO et al., 2019). To characterize such ST, the work of Mehren et al., (2018) has been considered. Mehren et al., (2018) empirically validated a two-dimensional model for ST competency for addressing environmental systems: one retrospective dimension (ST-OrgBeh) that covers issues related to the understanding of the system and its functioning (components and relationships), and a prospective dimension (ST-Action) that includes the ability to make forecasts and formulating appropriate actions, which is demonstrated by anticipating the possible effects of proposed actions and admitting the predictability constraint of complex systems. The first dimension reflects knowledge about the system, while the second one the application of such knowledge.

To sum up, this study aims to explore the ability of a group of primary pre-service teachers to develop FT related competencies including ST and agency through their involvement in diverse activities oriented to this end. The research questions are:

- RQ1. What level of ST and appropriation of the future related to FT is performed by primary pre-service teachers engaging in a sequence of activities about the origin of epidemics?
- RQ2. What dimensions related to FT do the activities facilitate?

## Methods

Participants in this study were 47 pre-service elementary teachers (PETs) in the fourth year of the Primary Education Degree at a Spanish university working in small groups of 3–5 (11 groups, A–K). Working in groups has shown benefits for developing students’ ST (Gray et al., 2014). In this case, the groups were chosen by the PETs. The study was carried out during the 2021/2022 academic year, and the first author was their teacher.

**Fig. 1:**
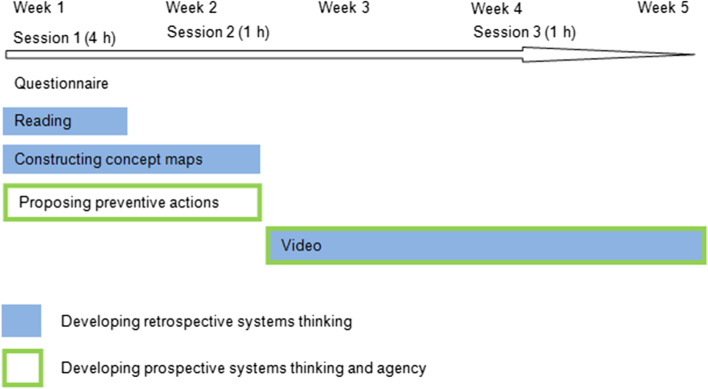
Sequence of activities (represented as a continuum because they required time commitment both in class and at home)

The activities focused on the origins of epidemics and pandemics and were part of a broader project about critical thinking on SSIs. The participants had not received previous instruction on pandemics. The sequence of activities is shown in Fig. 1, where the activities have been coloured depending on their purpose: the development of the knowledge of the system or retrospective ST (Mehren et al., 2018) or the development of prospective ST (Mehren et al., 2018) and agency. They were carried out in 3 sessions (6 h in total), but they were completed at home. Initially, PETs answered an open-ended questionnaire on pandemics. The purpose was to elicit their understanding, to pose the questions to be addressed and to stimulate interest about issues that they probably had not reflected on before, such as the prevention of pandemics. Then, PETs read science articles on the relationship between environmental problems and zoonoses that were provided to them, so that they developed a systemic perspective close to the “One Health” notion (FAO et al., 2019). Each group had to construct a concept map representing their understanding of the rise of epidemics based on provided information and to list preventive measures to avoid it. The teacher actively supported the PETs to establish relationships between the elements of the maps. She did not tell which elements or what relationships PETs should include, but she encouraged them to think about including new ones and to wonder if they were missing any. As a final activity, to get PETs more involved in the problem, each group prepared a 10–15-min video. The video was to be developed for the general public for the purpose of raising awareness of the increasing risk of epidemics and encouraging people to take action. It had two parts: in the first part (part a), each group had to explain the causes of and potential solutions for the rise of epidemics, using what they had learnt, and in the second one (part b), they had to choose one of the solutions to avoid future pandemics and propose a campaign around it.

### Data Collection and Analysis

The research is based on an interpretative study (Erickson, 1986) of qualitative data (written productions and video transcripts). The categories of analysis were initially established based on the theoretical perspectives about FT and ST (Hipkins, 2021; Jones et al., 2012; Levrini et al., 2021; Mehren et al., 2018). A constant comparative method (Lincoln & Guba, 1985) was used to analyse the data, which resulted in the adaptation and definition of diverse categories, as described below.

Data collection included students’ written productions (individual initial answers to the open-ended questionnaire and concept maps by groups) and the videos created by small groups. The research was approved by the university’s ethics board and participants signed an informed consent form. Being a teacher-research (Roth, 2007), the board demanded specific conditions that were fulfilled.

To address RQ1, individual written answers to these questions of the open-ended initial questionnaire were examined: “In your opinion, what increases the chances of pandemics such as COVID-19 appearing?” and “What can we do to prevent the appearance of another pandemic?.” They were not segmented to carry out the analysis (Chi, 1997), but researchers searched for occurrences of the dimensions related to ST, and the appropriation of the future (APP), which includes the “inside the system” perspective, anticipating the future and being an agent of change in it (Hipkins, 2021; Jones et al., 2012; Levrini et al., 2021; Mehren et al., 2018). The dimensions and levels are displayed in Table 1.

**Table 1:** Dimensions and levels for data analysis

Dimension	Level	Description
ST-Components	1	Components of only one “One Heath” sphere
	2	Components of two “One Health” spheres
	3	Components of the three “One Health” spheres
ST-OrgBeh	0	No cause-effect relationships
	1	Monocausal relationships
	2	Linear relationships
	3	Complex relationships
ST-Action	0	No cause-effect relationships
	1	Monocausal relationships
	2	Linear relationships
	3	Complex relationships
APP-Time	0	No time reference
	1	Time references
	2	References to the future
APP-Agent	0	No first-person reference
	1	Causes in first person
	2	Actions in first person

To characterize ST, the two-dimensional model of Mehren et al., (2018) was adapted.

ST-Components was a third dimension added to the proposal of Mehren et al., (2018), taking into account that one of the objectives of the activities was for PETs to broaden their views on the components involved in health towards a “One Health” notion. This dimension focuses on the components of the system (Hmelo-Silver et al., 2017) and corresponds to references to the three spheres (human, environmental and animal) of that approach (FAO et al., 2019).

ST-OrgBeh draws from “system organization and system behaviour” (Mehren et al., 2018), and characterizes the structure of the system and its behaviour (type of cause-effect relationships between the different parts). Monocausal relationships represent structures such as A causes B. Linear relationships correspond to chains (A causes B, B causes C), or to structures as A, B, and C act on X. Complex relationships refer to networks of chains, and feedback loops. In this work, it was necessary to add Level 0, as some productions did not show any cause-effect relationships.

ST-Action was adapted from “system-adequate intention to act” (Mehren et al., 2018). Its analysis is similar to that of ST-OrgBeh, but in relation to the effects of actions. Levels were adapted, including Level 0. When participants explained their proposal of actions referring implicitly to the effects of the action it was considered a “vague anticipation of effects” (Mehren et al., 2018, p. 701) and they were considered at Level 1. For example, Group A proposed to collect waste, but they explained it alluding to the effects indirectly: they explained why the waste can cause the transmission of diseases, so it can be inferred that collecting the waste would cause reducing the chances for such transmission, but they did not develop prognoses based on linear analysis of effects, and did not think about other effects: “It is important to say why we have to do this (..) There are more and more people in the world, so we generate more and more waste, and almost all waste finds its way into different ecosystems. So the animals are in contact with the waste. As we destroy ecosystems, animals are getting closer and closer to us, so they can pass these diseases on to us.”

APP-Time dimension encompasses the ability to anticipate events. Anticipating the future and approaching it are related to the ability to imagine the future, to see that the future is accessible through present actions and to see oneself as an agent (Levrini et al., 2021). The highest level includes references to possible futures. The intermediate level was established for temporal references to the past and/or present, which, while denoting the ability to move thinking through time, do not do so specifically with respect to the future*.*

APP-Agent dimension characterizes how participants see themselves as agents of the changes that they propose to reach the desirable future. It was analysed based on the use of the first person when stating actions. The use of the first person was a criterion used by Granit-Dgani et al., (2017) to assess the objective of environmental education as related to building personal and collective responsibility towards sustainability. The use of the first person was also highlighted in the study of Sass et al., (2021), who defined dimensions for action competency and developed an instrument for its assessment. An intermediate level was established for the case of using the first person, only when listing causes, as assuming the role of agents in the problem implicitly makes them agents in the solution.

One third of the transcripts were analysed independently by two researchers. Disagreements (15%) were discussed, and consensus was reached.

To address RQ2, the concept maps and videos elaborated by PETs were explored. System structure was analysed in the maps (Mehren et al., 2018; Rempfler, 2010). System structure consisted of the ratio of the sum of arrow chains (sets of three arrows in the same direction), converging and diverging branches (nodes to which two arrows are directed and/or from which two arrows depart) and loops (closed chains of arrows), and the number of map elements (Rempfler, 2010). The correspondence between the system structure and the levels of ST-OrgBeh was established by Mehren et al., (2018).

As the objective of the video-campaign activity was to get the participants more involved in the problem, the discourse in the video was analysed and categorized for the dimensions related to action, the prospective dimensions. Parts a and b of the videos were analysed separately to make comparisons. For doing so, one-third of the transcripts were analysed independently by two researchers. The first author analysed all other transcripts.

## Results

### Pre-service Elementary Teachers’ Performance on Futures Thinking–Related Dimensions

**Fig. 2:**
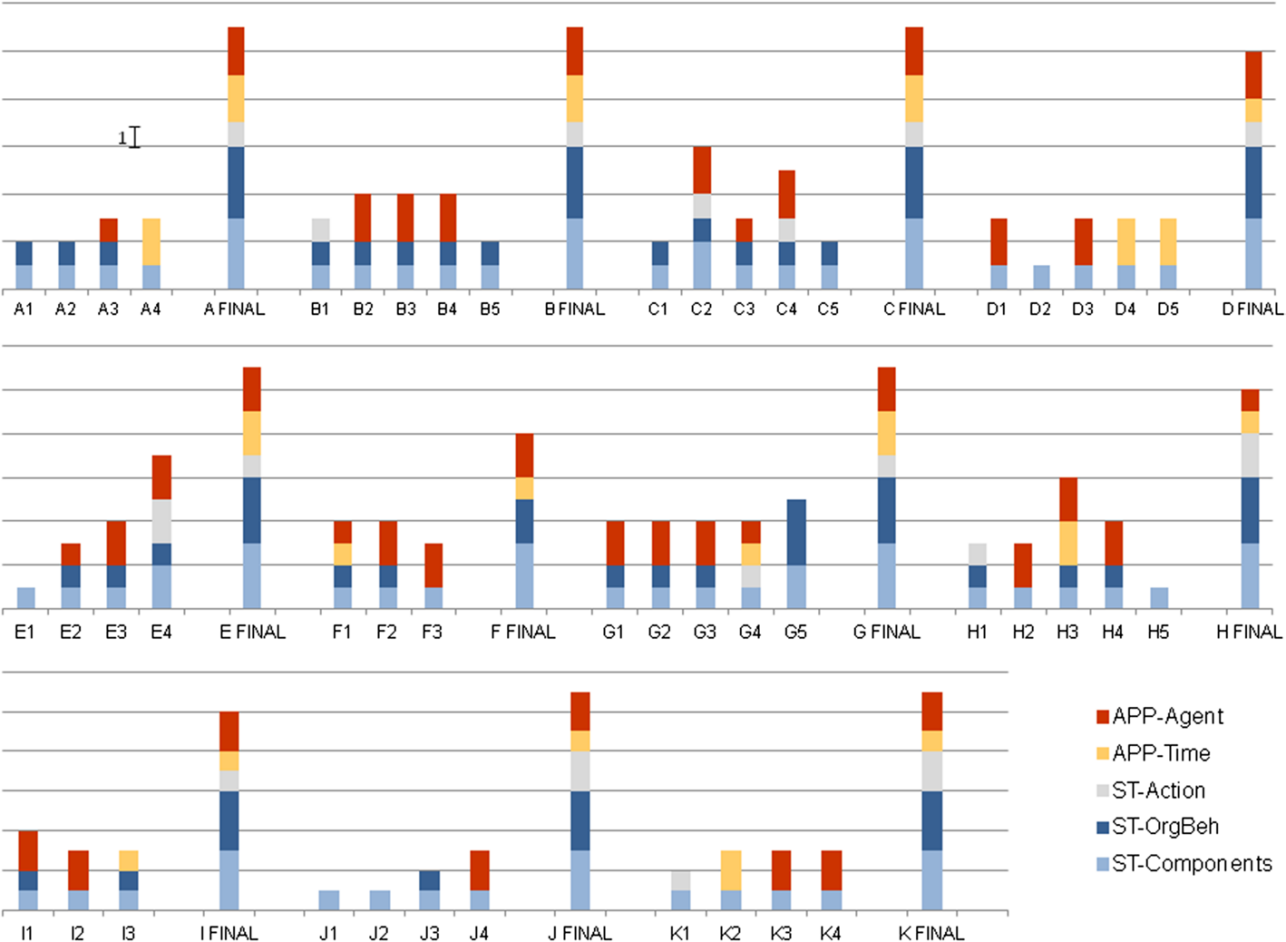
Levels (it is indicated in the figure which interval in *y* axis corresponds to one level) achieved in the dimensions related to FT. A1, A2… refers to individual group A members at the initial questionnaire and A FINAL refers to group A at the final activity video)

Figure 2 shows the levels achieved for each dimension by the PETs in their individual responses to the open-ended questionnaire at the beginning of the sequence and in the final group video.

As Fig. 2 shows, FT-related competences were developed by all groups, so that the levels of the dimensions were higher in the final product than in the initial individual responses of its members. Table 2 displays the average values obtained for each dimension at the initial and final moments.

**Table 2:** Average values for the dimensions

Dimension	Initial (*N* = 47)	Final (*N* = 11)
ST-Components (1–3)	1.06	3
ST-OrgBeh (0–3)	0.60	2.91
ST-Action (0–3)	0.17	1.18
APP-Time (0–2)	0.28	1.45
APP-Agent (0–2)	1.04	1.91

The ST-Components dimension improved significantly. The PETs moved from a perspective in which they mostly considered only one or a few elements of a single sphere (70%) — mainly human (59%) — to a situation in which all groups considered multiple elements of the three spheres as interrelated. Moreover, they did so by establishing cause-effect relationships between them. The presence of the diverse elements considered for the final ST-OrgBeh is shown in Table 3.

**Table 3:** Presence of “One Health” components, linear chains, links between chains, and feedback loops in the final activity (video)

Group	Number of “One Health” components	Linear chains	Links between chains	Feedback loops	ST-OrgBeh
A	3	≥ 3	≥ 3		3
B	3	≥ 3	1	1	3
C	3	≥ 3	≥ 3		3
D	3	≥ 3	1	1	3
E	3	≥ 3	≥ 3		3
F	3	1			2
G	3	≥ 3	≥ 3		3
H	3	2	1	1	3
I	3	2	1		3
J	3	≥ 3	≥ 3		3
K	3	≥ 3	≥ 3		3

Seven groups established complex cause-effect networks by establishing linear chains and connections between them. See the following excerpts from group K as an example of how they made these linear chains and connections.
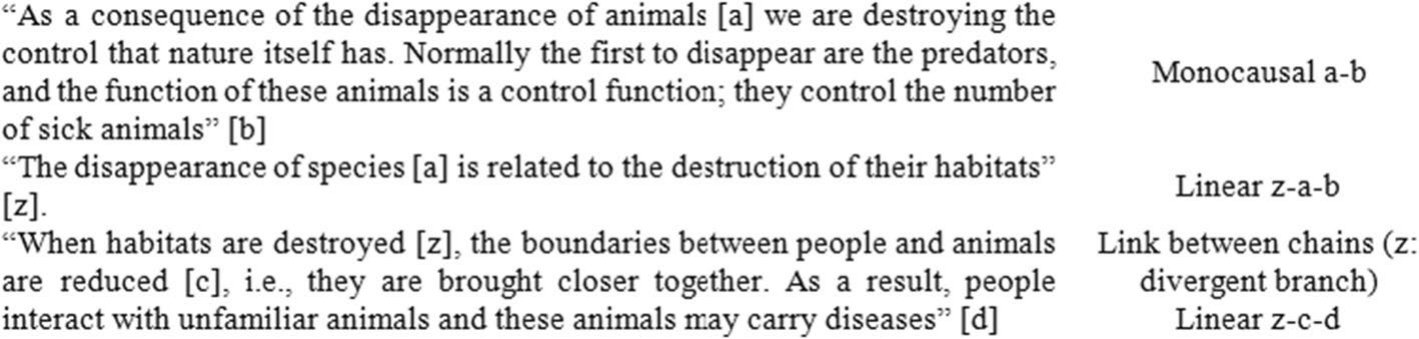


Three groups made feedback loops. One of them was group D:
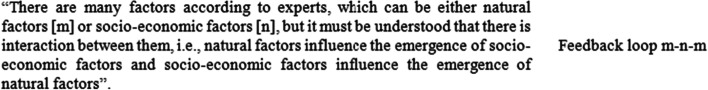


Group F was the only one categorized in level 2 as they just explained one linear chain.

However, monocausal relationships formed the majority of specifications by the end in the prospective dimension ST-Action. No group created complex networks, and only three came up with a more elaborate linear relationship than the monocausal one. The next excerpt taken from group K shows a linear relationship corresponding to ST-Action 2.
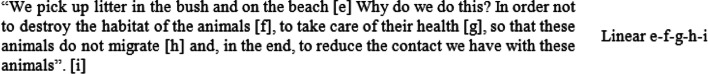


Two other groups resorted to linear relationships in retrospect to justify solutions and one group did not relate their proposals to any effects, as 85% of PETs had initially done.

The dimensions related to future approaches increased in level in the final group work compared to the individual work. Only 5 PETs out of 47 referred to the future (APP-Time 2) initially, while 5 of the 11 groups did so. In terms of seeing oneself as an agent of change to the future, the initial situation was better than that for the other dimensions, with 47% of PETs expressing actions to prevent pandemics in the first person (APP-Agent 2). 10 groups were at this level.

### Dimensions of Futures Thinking Fostered by the Activities (RQ2)

**Fig. 3:**
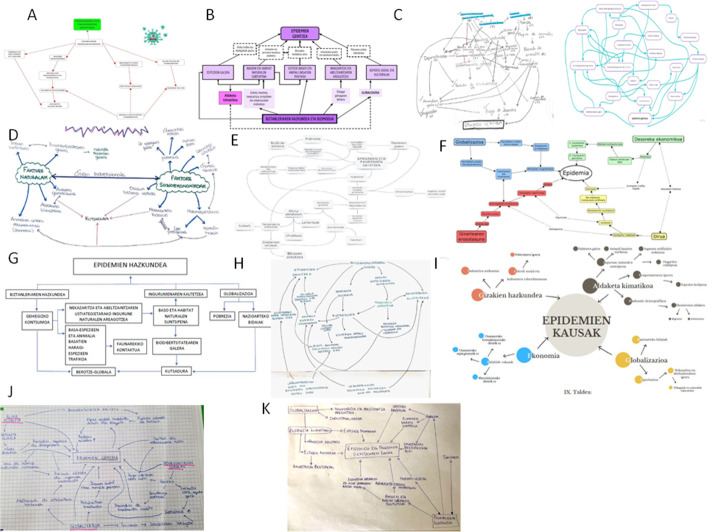
The visual appearance of networks about the increase in epidemics for each group (**A**–**K**)

Figure 3 shows an image of the conceptual maps elaborated by the groups.

**Fig. 4:**
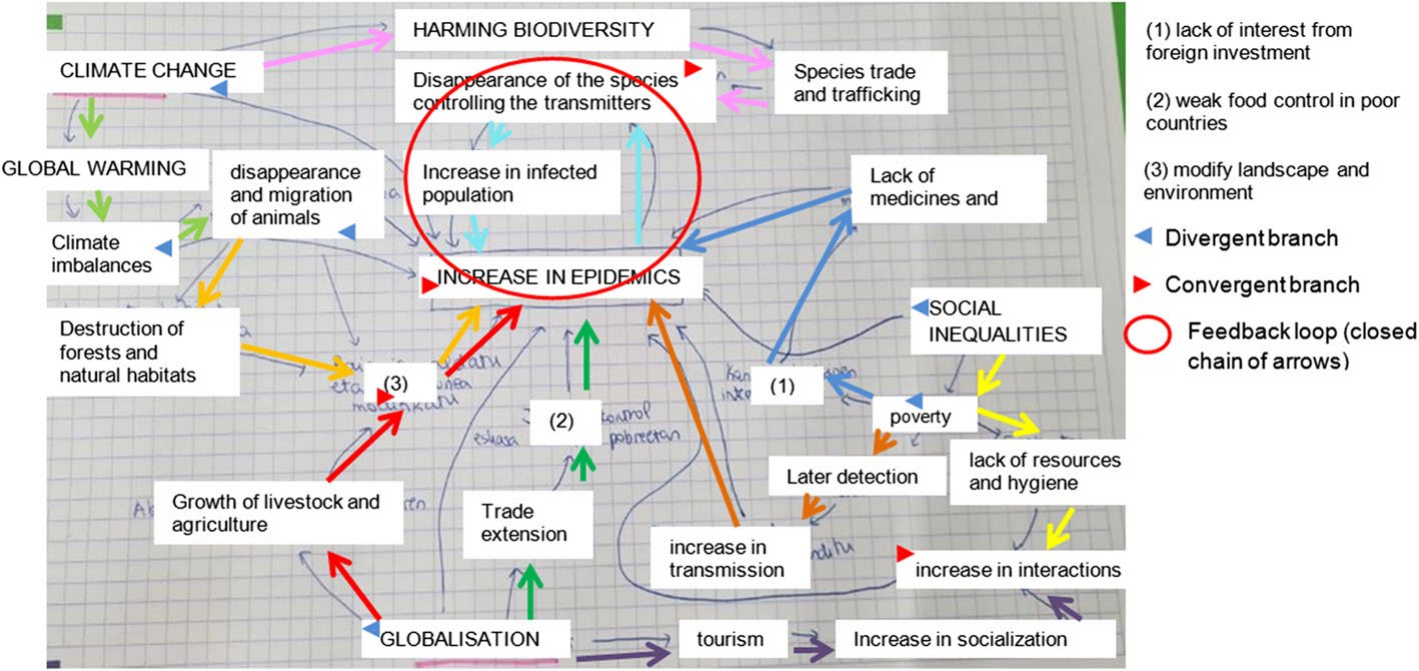
Linear chains, branches and feedback loops in group J´s map (translated to English)

Figure 4 shows the analysis of the map of Group J as an example, given that it contains all types of elements. Each arrow chain (sets of three arrows in the same direction) is represented in one colour (total: 10) Converging and diverging branches (nodes to which two arrows are directed and/or from which two arrows depart) are represented by red and blue triangles, respectively (4 and 6 in total), and the only feedback loop (a closed chain of arrows) is represented as a circle.

Table 4 shows the results of the analysis of the maps.

**Table 4:** Presence of “One Health” components, linear chains, branches, and feedback loops in the conceptual maps

	Number of elements	Number of “One Health” components	ST-Components	Chains	Branches		Feedback loops	System structure	ST-OrgBeh
					Convergent	Divergent			
A	12	3	3	1	4	2	0	0.6	3
B	16	3	3	5	5	4	5	1.1	3
C	20	3	3	> 6*	8	9	0	> 1	3
D	18	3	3	1	1	2	3	0.4	2
E	25	3	3	6	6	8	0	0.8	3
F	23	3	3	8	5	5	1	0.8	3
G	14	3	3	4	3	4	0	0.7	3
H	16	3	3	5	5	6	1	1.1	3
I	27	3	3	2	1	7	0	0.4	2
J	24	3	3	10	4	6	1	0.9	3
K	19	3	3	6	2	7	2	0.9	3

The 11 groups included elements from all three spheres (human, environmental, animal) as shown in Fig. 3 and Table 4. All of them established at least one linear chain, and convergent and divergent branches. There were a total of 60 divergent branches and 44 convergent branches. Six groups constructed at least one feedback loop.

**Fig. 5:**
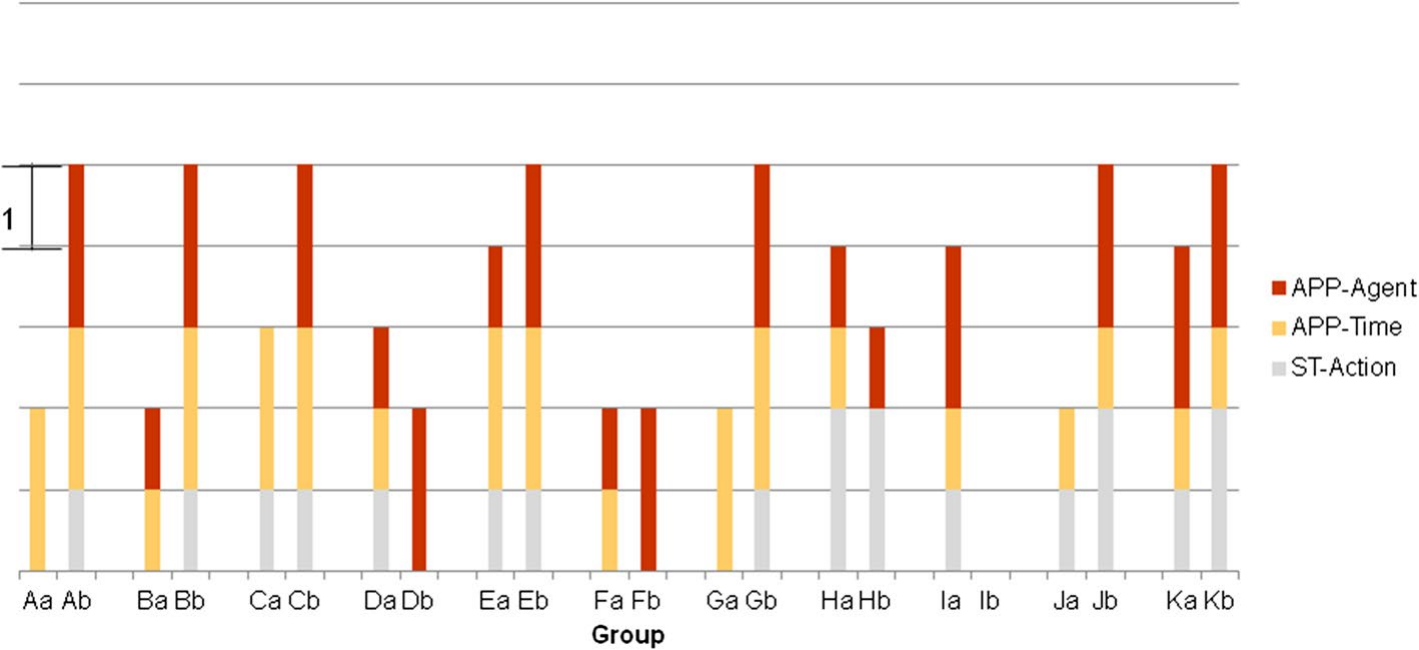
Levels (it is indicated in the figure which interval in y axis corresponds to one level) of the prospective dimensions in the two parts of the video

Figure 5 shows the level reached by each group in the prospective dimensions in the two parts of the video: (a) explanation of causes and solutions to the problem, and (b) campaign.

**Fig. 6:**
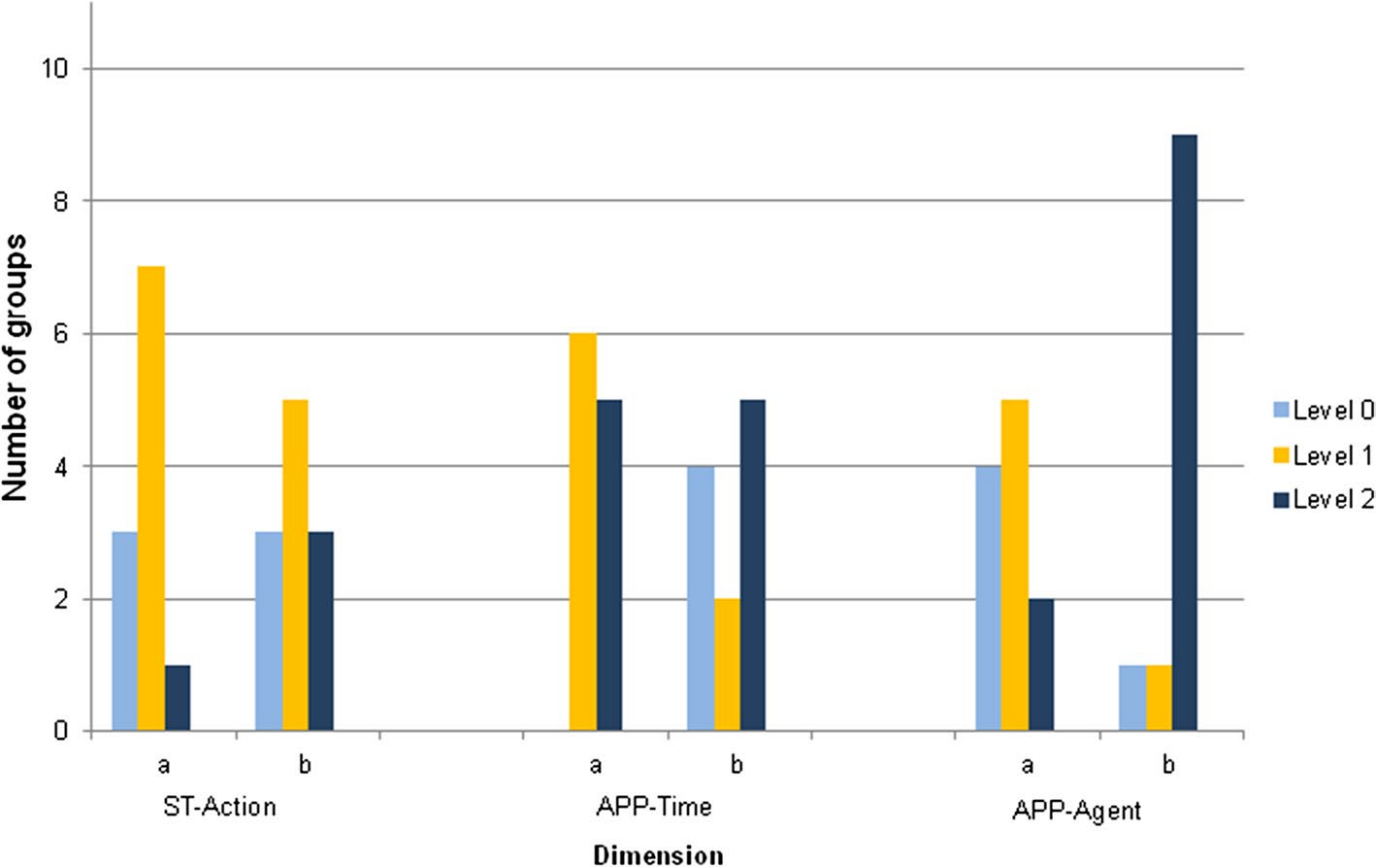
Number of groups at each level of the prospective dimensions in the two parts of the video

Figure 6 shows the number of groups at each level of the prospective dimensions in the two parts of the video.

Despite the diversity of the performance of the various groups (Fig. 5), it can be seen in Fig. 6 that, overall, the differences were small in the case of the results between the parts of the video for ST-Action (two groups more in level 2 in part b than in part a) and for references to the future in APP-Time (references to the past decreased in part b). However, there were remarkable differences in the case of APP-Agent, with an increased use of the first person in the campaign. In fact, the groups, even referring to the same aspects, changed the language. For example, group A in part a referred to the causes as follows: “When forests disappear, animals have to migrate and come closer to people. This causes zoonoses or diseases transmitted by animals, as in the case of COVID-19,” whereas in the campaign (part b) they said: “We generate more and more waste, and almost all waste reaches the different ecosystems […] As we destroy ecosystems, animals are closer and closer to us, so they can pass these diseases to us and that can be an example of generating a pandemic.”

## Discussion

The pandemic of COVID-19 has highlighted the need to develop a citizenry with skills to analyse complex situations, as well as to anticipate potential problems by developing forward-looking thinking, which promotes action. Current educational perspectives (OECD, 2018) acknowledge the relevance of these skills; however, research on their development in SSI instruction is still scarce.

In this study, a sequence of activities was designed and implemented to promote ST, the ability to anticipate the future and the perspective of being agents of change, in a group of PETs in the context of an increase in zoonotic infectious diseases (Jones et al., 2008).

Regarding the first research question (RQ1), the results show that ST was developed, especially in its components and behaviour dimensions. Concerning the organizational/structural part of the system, PETs initially showed a limited perspective, which is aligned with a previous study (authors, 2021). This placed PETs at the lowest level of ST according to all models (Hmelo et al., 2017; Hmelo-Silver & Pfeffer, 2004; Mehren et al., 2017), that describe low level ST as thinking that is focused on the structural components, particularly the visible ones, which in the case of this study would correspond to those of the human dimension.

Students in other studies on ST in natural systems had shown a better performance in ST (e.g., Hmelo-Silver et al., 2017; Mambrey et al., 2022; Snapir et al., 2017). With the participants in this research being adult undergraduates, higher levels might be expected. Nevertheless, also, the trainee teachers in the Palmberg et al., (2017) study showed large deficiencies in ST: three quarters of the 424 PETs showed no evidence of ST when relating species identification, biodiversity, and sustainable development. This suggests that there is a leap in the demand for ST between contexts that only imply one sphere such as biology, and holistic contexts, such as socio-environmental or socio-health contexts, as required by the “One Health” vision (FAO et al., 2019).

In the final activity, i.e., the video, all groups considered multiple, interrelated elements of the three spheres. For instance, they moved from focusing on aspects related to health measures in the current pandemic situation, such as the influence of travel, to other factors, both social (e.g., migration of people and accumulation of people in cities), environmental (status and functioning of ecosystems, climate change), and animal (migration of animals, accumulation in farms and markets), and their consequences. Furthermore, they did so by establishing cause-effect relationships between them, which positioned them as expert learners (Hmelo-Silver & Pfeffer, 2004).

The PETs justified the interrelationships through mechanisms and were able to explain various phenomena related to the problem under study that they had not initially described, e.g., the mechanisms leading to transmission between animals and from animals to humans. Being able to refer to the components of the system, the mechanisms, and the phenomenon they produce — as well as relating these elements — corresponds to the highest level of ST according to Hmelo-Silver et al., (2017).

The groups established cause-effect relationships of linear and complex type. Establishing cause-effect relationships is in fact, a cognitively demanding competence in which students have been shown to have difficulties (Levrini et al., 2021; Mambrey et al., 2022) and that it has been the subject of recent proposals (Melles et al., 2021). However, the groups did not establish complex cause-effect relationships when considering actions, i.e., when thinking prospectively. This may be related to the cognitive demand of the prospective dimension of ST regarding to the other, since as Mehren et al. (2017) pointed out, components and behaviour of the system relate to knowledge of the system, whereas prospective thinking relates to the application of that knowledge. It would be relevant to explore how to engage participants in the articulation of this dimension in future research.

Regarding the second research question (RQ2), to develop understanding of the system, the PETs had acquired a variety of information and had made mind maps. The elaboration of the maps showed that this process helped participants in the articulation of diverse elements retrieved from the information provided. Nine groups performed at the highest level in the corresponding dimensions of ST, constructing maps with chains of arrows, divergent and convergent branches, and feedback loops. They performed this task in groups; previous studies have underlined the importance of group work for ST (Gray et al., 2014). The teacher actively intervened to support PETs in the process of making connections between the elements of the maps; thus, teacher guidance and scaffolding could be crucial for developing this ability. Feedback loops are of higher complexity than the other elements in the system structure (Rempfler, 2010). Only six groups represented them in the maps and three of those constructed one in the final activity. Only groups that had constructed a map with links between chains or feedback loops were able to show these elements at the end. Although the data are limited, it seems that having made maps showing elements of ST acted as a necessary, but not sufficient, condition.

For the participants to take ownership of the issue and develop the prospective dimensions, they carried out a campaign related to the implementation of an action. The results show that in the campaign the PETs mobilized, above all, the sense of being inside the system and the sense of being agents of change, making use of the first person.

Science lessons specifically those addressing SSI are uniquely placed to foster ST, FT, and action competence as OCDE (2018) and science educators (Hodson, 2003; Jones et al., 2012; Levrini et al., 2021) suggest, to succeed in shaping agents of change for a better future. To do so, teachers would benefit from being provided with a set of strategies and resources with evidence-based effectiveness. This study contributes to the body of research that has primarily addressed strategies for developing aspects of ST, FT and action competence in science education (e.g., Ben-Zvi Assaraf & Knippels, 2022; Hipkins, 2021; Jones et al., 2012; Levrini et al., 2021).
